# Clinical Relevance of Isoagglutinin Rebound in Adult ABO-Incompatible Living Donor Liver Transplantation

**DOI:** 10.3390/jpm11121300

**Published:** 2021-12-05

**Authors:** Wei-Chen Lee, Chen-Fang Lee, Tsung-Han Wu, Hao-Chien Hung, Jin-Chiao Lee, Yu-Chao Wang, Chih-Hsien Cheng, Ting-Jung Wu, Hong-Shiue Chou, Kun-Ming Chan

**Affiliations:** 1 Division of Liver and Transplantation Surgery, Department of General Surgery, Chang-Gung Memorial Hospital, Linkou 33357, Taiwan; lee5310@cgmh.org.tw (C.-F.L.); wutsunghan@gmail.com (T.-H.W.); mp0616@cgmh.org.tw (H.-C.H.); b9302012@cgmh.org.tw (J.-C.L.); awuang726@gmail.com (Y.-C.W.); chengcchj@cgmh.org.tw (C.-H.C.); wutj5056@cgmh.org.tw (T.-J.W.); chouhs@cgmh.org.tw (H.-S.C.); chankunming@cgmh.org.tw (K.-M.C.); 2 Department of Surgery, College of Medicine, Chang-Gung University, Taoyuan 33303, Taiwan

**Keywords:** ABO-incompatibility, liver transplantation, rituximab, bortezomib, case series

## Abstract

ABO-incompatible (ABO-I) living donor liver transplantation (LDLT) can be performed successfully. However, anti-ABO isoagglutinin rebound may cause antibody-mediated rejection (AMR) and graft loss. The risk threshold of isoagglutinin rebound is still not defined. 76 ABO-I LDLT recipients were divided into group A (*n* = 56) with low isoagglutinin titers (<1:256), and group B (*n* = 20) with high isoagglutinin titers (≥1:256), at initial assessment for liver transplantation. The last 12 patients in group B received a modified desensitization regimen by adding bortezomib to deplete plasma cells. Six (10.7%) patients in group A and 10 (50.0%) patients in group B had postoperative isoagglutinin rebound (*p* < 0.001). Three patients (5.54%) in group A and two patients (10%) in group B developed clinical AMR (*p* = 0.602). The cutoff value of postoperative isoagglutinin rebound to cause clinical AMR was ≥1:1024. Among the 12 patients in group B with bortezomib administration, isoagglutinin rebounded up to 1:128 only, and no clinical AMR occurred. In conclusion, the patients with high isoagglutinin titers had a higher rate of postoperative isoagglutinin rebound. Isoagglutinin rebound ≥1:1024 is risky for developing clinical AMR. Adding bortezomib into the desensitization regimen may mitigate isoagglutinin rebound, and avoid clinical AMR.

## 1. Introduction

Liver transplantation is the only effective treatment for patients with acute liver failure or end stage of liver diseases [1,2]. Liver transplantation is also indicated for hepatocellular carcinoma (HCC) if the tumors meet certain criteria [3,4,5]. In the Barcelona Clinic Liver Cancer (BCLC) staging and therapeutic strategies, liver transplantation is suggested for early-stage HCC with portal hypertension to yield the best outcomes [6]. Clearly, indications of liver transplantation are expanded from benign diseases to liver cancer. Hence, the demand of liver grafts for transplantation is increased and shortage of liver grafts for transplantation may be worse than before.

Living donor liver transplantation (LDLT) is an option when deceased liver allografts are not available. Currently, LDLT is an alternative operation in Asian countries where deceased liver donation is always in short supply, and is also an emerging operation in western countries [7]. Most countries regulate that living donors must be emotionally intimate individuals; as such living organ donation is restricted to within fifth degree of intimate relatives in Taiwan. Therefore, LDLT crossing the blood-type barrier becomes an issue to overcome, since most families are small in size, and eligible members of the family are limited. The liver is recognized as an immune privilege organ and ABO-incompatible (ABO-I) liver transplantation has been performed for several decades [8]. Although the outcome of ABO-I liver transplantation was not particularly good in the 1990s, the results of ABO-I liver transplantation were much improved after rituximab was introduced to organ transplantation [9]. Currently, there are several methods to decrease anti-A/B isoagglutinin titers in recipients due to undergo ABO-I LDLT, including rituximab infusion, intravenous immunoglobulin, anti-A/B antibody immune-adsorption, plasmapheresis, plasma exchange, etc. [10,11,12,13]. Under such preparation regimens, ABO-I LDLT can be performed successfully, and leads to similar results of ABO-compatible (ABO-c) LDLT [13,14,15].

However, clinical antibody-mediated rejection (AMR) may occur in ABO-I liver transplantation and cause graft loss [9,16]. AMR is related to anti-A/B antibody rebound. Who is likely to experience antibody rebound is still not clear. Inferentially, the recipients with high titers of anti-A/B antibody may be at risk of experiencing antibody rebound and AMR. Herein, we divided our patients according to low or high isoagglutinin titers prior to liver transplantation in order to clarify the clinical relevance of isoagglutinin titers prior to liver transplantation. In addition, we also wished to speculate the level of postoperative antibody rebound on AMR in ABO-I LDLT.

## 2. Materials and Methods

*Patients.* Seventy-six adult patients with ABO-I LDLT at Chang-Gung Memorial Hospital were included. The patients were divided into group A patients, whose anti-A/anti-B isoagglutinin titers were < 1:256, and group B patients, whose isoagglutinin titers were ≥1:256. Group A was defined as the low-titer group, and group B was defined as the high-titer group. All donors were within third-degree relation to the recipients. This study conformed to the ethical guidelines of the 2000 Declaration of Helsinki, and was approved by institutional review board of Chang-Gung Memorial Hospital (IRB No. 20171223BO).

*Definition of high isoagglutinin titer.* The definition of high isoagglutinin titer was defined as anti-blood type isoagglutinin titer ≥1:256, which was referenced from blood transfusion. In whole-blood transfusion, some group O donors had very high titers of anti-A or anti-B isoagglutinin titers, which caused hemolysis. Donors with titers ≥1:256 were determined to be dangerous donors [17].

*Determination of isoagglutinin titers.* Anti-A/B isoagglutinin detection was determined by MeDipro antibody screening cell kit, and the procedure was conducted according to the manufacturer’s instructions (Formosa Biomedical Technology Corp., Taipei, Taiwan). Briefly, 100 uL fresh serum was added into 50 uL of A cells or B cells of the kit, mixed, and centrifuged at 3400 rpm for 15 min. Anti-A/B isoagglutinin was positive when cells were aggregated or lysed. The titers of anti-A/B isoagglutinin were determined by subsequent 2-fold dilution of serum.

*Desensitization regimen for ABO-I liver transplantation.* The regimens reducing isoagglutinin titers to undergo ABO-I LDLT were according to our previous study [14]. In brief, if isoagglutinin titers were ≤1:64, liver transplantation was directly undergone, and B-cells were depleted by rituximab (375 mg/m^2^) on postoperative day (POD) one. If anti-blood type isoagglutinin titers were >1:64, desensitization was prepared by intravenously rituximab (375 mg/m^2^) two to three weeks before liver transplantation, followed by plasma exchange one week before transplantation to achieve anti-A or anti-B antibody titers ≤1:64.

**Figure 1:**
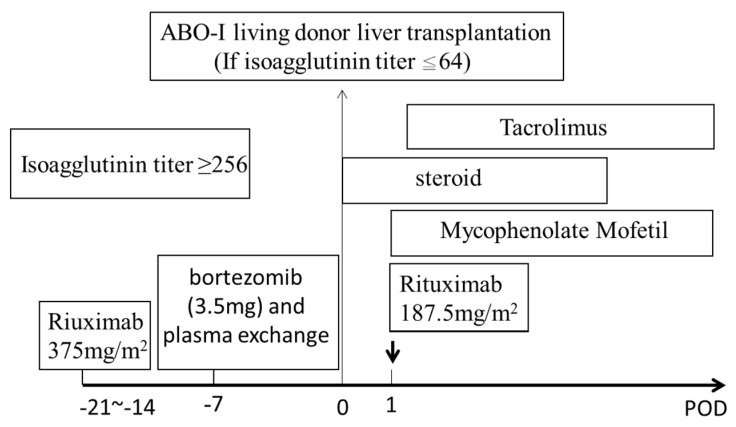
Modified desensitization regimen for preparing ABO-I living donor liver transplantation. If isoagglutinin titers were ≥256, desensitization was prepared by intravenously rituximab (375 mg/m^2^) two to three weeks before liver transplantation, bortezomib (1.3 mg/m^2^) to deplete plasma cells, and several courses of plasma exchange at one week before transplantation.

*Modified desensitization regimen for ABO-I liver transplantation.* For the patients with high isoagglutinin titers, the desensitization regimen was modified by adding bortezomib (1.3 mg/m^2^) to deplete plasma cells (Figure 1). When the isoagglutinin titers were ≥1:256, rituximab was administered two to three weeks before transplantation to deplete B-cells, and bortezomib was administered one week before transplantation to deplete plasma cells, followed by plasma exchanges to achieve isoagglutinin titers ≤1:64. The courses of plasma exchange depended on achievement of isoagglutinin titers ≤1:64.

*Definition of isoagglutinin rebound.* All of the patients’ isoagglutinin titers were ≤1:64 prior to liver transplantation. After transplantation, an isoagglutinin titer >1:64 was defined as isoagglutinin rebound.

*Postoperative following up and management.* After transplantation, anti-blood type isoagglutinin titers were measured every other day in the first week, every week in the first month, and then every three months. The isoagglutinin titer would be measured if it was indeed necessary. While isoagglutinin titers rebounded to ≥1:256, plasma exchanges were prepared to perform for isoagglutinin titer reduction.

*Immunosuppressive regimen.* The immunosuppressive regimen was consisted with steroid, tacrolimus, and mycophenolate mofetil. During operation, methylprednisolone, 500 mg, was given intravenously. Postoperatively, methylprednisolone was tapered from 200 mg/day to 40 mg/day over five days, and discontinued within three months after the operation. Mycophenolate mofetil (MMF; 1 g/day) was given orally from POD 1. The administration of tacrolimus was delayed and started orally on POD 2 or 3 while renal function returned. The therapeutic trough levels (7–10 ng/mL) of tacrolimus were achieved within 7 days after transplantation.

*Following up of liver function.* Aspartate aminotransferase (AST), alanine aminotransferase (ALT), bilirubin, and prothrombin time were measured every 6 h in the first week, every 12 h in the second week and then every day/every other day in the following hospitalized days.

*Imaging studies.* After transplantation, blood flow of the liver was measured by Doppler ultrasonography every other day in the first week. Computed tomography (CT) was performed at the end of the first month. Then, abdominal ultrasonography and CT were performed every three months. However, if abnormal liver function was found, liver Doppler ultrasonography would be performed first to evaluate liver parenchyma, biliary tracts, and blood flow of the hepatic artery and portal vein. If abnormal liver blood flow or dilated bile ducts were found, abdominal CT was performed to confirm the findings of liver ultrasonography.

*Diagnosis of clinical T-cell mediated and antibody-mediated rejection (AMR).* To prevent the complication of liver biopsy, acute rejection was diagnosed by clinical manifestations and biochemical abnormalities of hepatic enzymes. T-cell mediated cellular rejection was suspected when AST and ALT were increased more than 30 u/L over the previous day, as mentioned by Kasahara et al. [18]. Acute clinical AMR was suspected by surged serum levels of AST and ALT and markedly decreased flow of the portal vein [19]. The clinical manifestations of AMR included rapid increase of AST and ALT, hyperbilirubinemia coagulopathy, ischemic cholangiopathy, graft dysfunction, and liver failure, as described by Egawa et al. [9,20] Liver biopsy was reserved for those patients whose persistent abnormal liver function could not be differentiated by clinical manifestations, and whose coagulation status was allowed for liver biopsy.

*Treatment of biliary stricture.* If biliary stricture was suspected, endoscopic retrograde cholangiographic (ERC) papillotomy with stenting was the first choice of treatment. If ERC stenting failed to dilate the biliary stricture, percutaneous transhepatic cholangiography was performed to pass a catheter through the stricture site for dilating and stenting biliary stricture.

*Biostatistics.* Unpaired Student’s t-tests were used to analyze continuous variables. Categorical variables were analyzed by either Chi-squared test or Fisher’s exact test. All pairwise multiple comparisons were done using the Holm–Sidak method. Meanwhile, the survival rates were calculated using the Kaplan–Meier method, and compared between groups using the log-rank test. The cutoff value of postoperative isoagglutinin titers to cause AMR was assessed by receiver operating characteristic (ROC) curve. The statistical analyses were all performed with SigmaPlot 14.0 software for Windows (Systat Softwave, Inc., San Jose, CA, USA). *p* < 0.05 was considered statistically significant.

## 3. Results

### 3.1. Patients

A total of 76 undergoing ABO-I LDLT in our institute from 2006 to 2016 were included in this study. The median (interquartile, IQ) isoagglutinin titer of all 76 patients was 1:128 (1:64 to 1:256), with a range from 1:8 to 1:2048. The patients were divided into group A patients (*n* = 56) with isoagglutinin <1:256, and group B (*n* = 20) patients with isoagglutinin titers ≥ 1:256. The patients in group B were above the 75th percentile of isoagglutinin titers. Hepatitis B-related diseases (41/76, 53.9%) were the most common etiologies, followed by hepatitis C (19.7%), and primary biliary cirrhosis (6.6%). Among the patients, 37 (48.7%) patients had hepatocellular carcinoma. The median (IQ) ages for group A and B patients were 56.0 (50.5–60.5) and 53.4 (48.3–57.8) years, respectively (*p* = 0.337). The median (IQ) MELD scores were 13 (9–18) for group A patients, and 16.5 (10–19.5) for group B patients, respectively (*p* = 0.281, Table 1).

### 3.2. Pre-Operative Isoagglutinin Titers and Plasma Exchange

The acceptable isoagglutinin titer on performed adult ABO-I LDLT was ≤1:64 in this study. Plasma exchange was performed for the patients whose isoagglutinin was >1:64. In group A, only 11 of 56 (19.6%) patients needed plasma exchange to achieve isoagglutinin titers ≤1:64. In group B, 17 of 20 (85%) patients needed plasma exchange to achieve isoagglutinin titers ≤1:64, excluding 3 patients with isoagglutinin titers ≤1:64 after rituximab administration. The median (IQ) courses of plasma exchange for group A patients were 0 (0-0), compared with 3 (2.3–4.5) for group B patients (*p* < 0.001).

### 3.3. Postoperative Isoagglutinin Rebound

**Figure 2:**
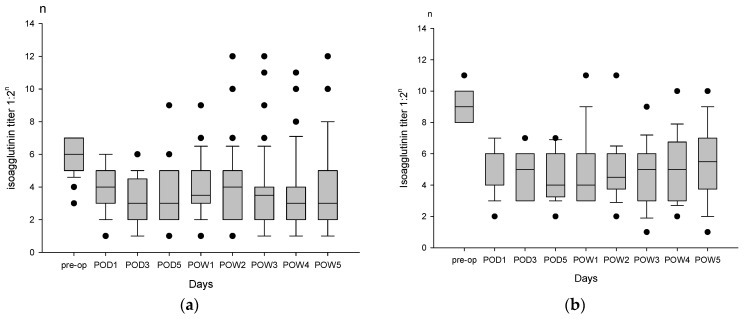
Isoagglutinin titers at pre-operation and post-operation within the first five weeks after liver transplantation. (**a**) Six of 56 patients in group A had postoperative isoagglutinin rebound (titers ≥ 1:128). Among the six patients, the rebound of isoagglutinin titers was up to 1:128 in two patients, 1:512 in one patient, 1:1024 in one patient, and 1:4096 in two patients. (**b**) 10 of 20 patients in group B had isoagglutinin rebound. The rebound of isoagglutinin titers was up to 1:128 in seven patients, 1:256 in one patient, and 1:2048 in two patients.

It was a critical issue whether isoagglutinin would rebound after operation in ABO-I liver transplantation. In this series, 16 of 76 patients (21.1%) had postoperative isoagglutinin rebound, including 6 (10.7%) patients in group A, and 10 (50%) patients in group B (*p* < 0.001). Among six patients in group A, isoagglutinin rebound was up to 1:128 in two patients, 1:512 in one patient, 1:1024 in one patient, and 1:4096 in two patients (Figure 2a). The isoagglutinin rebound began on POD 5 or 7. Among 10 patients in group B, isoagglutinin rebound was up to 1:128 in seven patients, 1:256 in one patient, and 1:2048 in two patients (Figure 2b).

**Figure 3:**
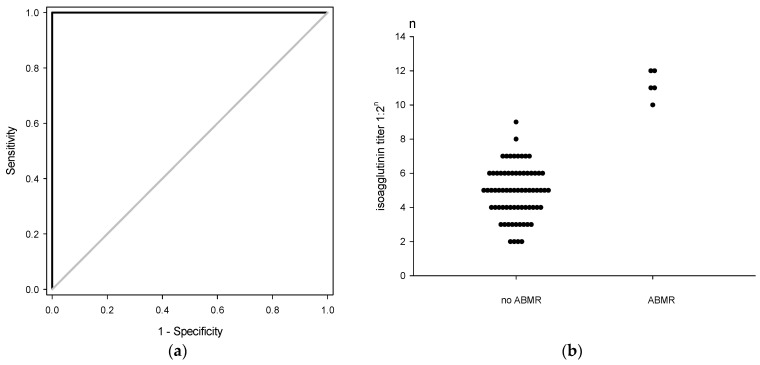
The threshold of postoperative isoagglutinin titer to cause AMR. (**a**) ROC curve of isoagglutinin titer to cause AMR. The best cutoff value of postoperative isoagglutinin rebound to cause clinical AMR was 1:1024 with 1.00 (95% CI: 0.48–1.00) of sensitivity and 1.00 (95% CI: 0.95–1.00) of specificity. (**b**) Dot histogram of isoagglutinin titer to cause AMR. In total, 16 of 76 patients (21.1%) had postoperative isoagglutinin rebound (≥1:128). Five of them developed AMR, whose isoagglutinin rebound was ≥1:1024.

The three patients in group A with isoagglutinin titers ≥1:1024 suffered from clinical AMR with ischemic biliary stricture, repeated biliary infections, and died of sepsis on POD 259, 285, and 306, respectively. For the two patients in group B with isoagglutinin titers ≥1:2048, one patient had hyperacute AMR with massive hepatocyte necrosis, and died of graft failure on POD 14, and the other one had ischemic biliary stricture, repeated biliary infections, and died on POD 409. In total, the complication of clinical AMR were three (5.4%) patients in group A and two (10%) patients in group B (*p* = 0.602). By ROC curve, the best cutoff value of postoperative isoagglutinin rebound to cause clinical AMR was 1:1024 (Figure 3a). All of the patients complicated with clinical AMR in this study had isoagglutinin titer rebounded to ≥1:1024 (Figure 3b).

### 3.4. Biliary Complications

**Figure 4:**
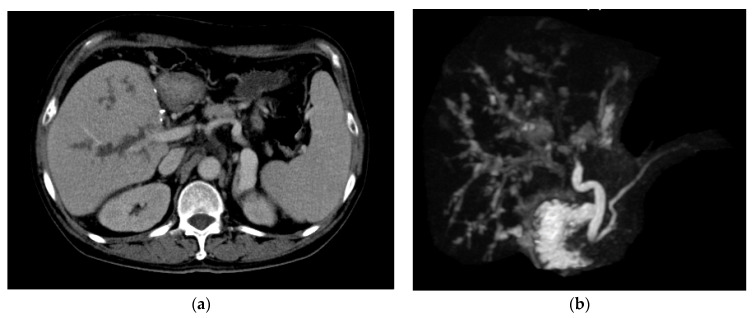
A representative of imaging study in AMR with ischemic biliary stricture. In this representative, (**a**) CT shows dilated biliary tract with irregular lining. (**b**) Magnetic resonance cholangiopancreatography shows multiple saccular dilatations of the biliary tract.

Biliary complication included biliary stenosis at anastomosis sites and ischemic biliary stricture at non-anastomosis sites. Ischemic biliary stricture was recognized as a clinical manifestation of AMR (Figure 4a,b). A total of 23 (41.1%) patients in group A had biliary complications. Of these, 20 of them were biliary stenosis at anastomosis sites, and three had ischemic biliary stricture. A total of 8 patients (40%) in group B had biliary complications. Of these, 7 had biliary stenosis at anastomosis sites, and one had ischemic biliary stricture. The biliary complication rates between group A and B patients were not different (*p* = 0.856). The ischemic biliary stricture rates were 5.4% in group A, compared with 5% in group B, respectively (*p* = 1.000).

### 3.5. Post-Transplantation Survival Rate

**Figure 5:**
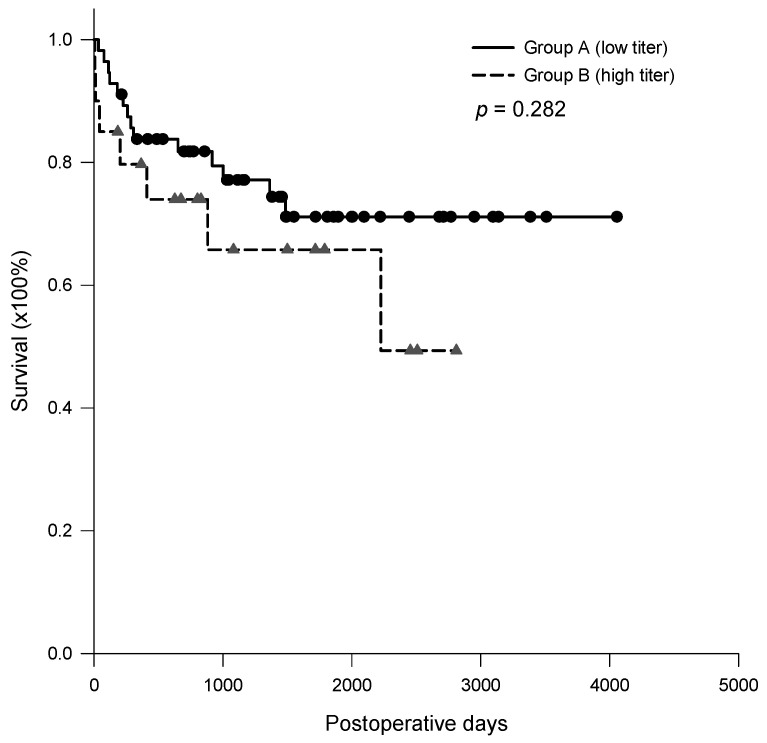
Kaplan–Meier survival curves for the patients in group A and B. The 6-month, 1-, 3- and 5-year graft and patient survival were 91.1%, 83.8%, 77.1%, and 71.1% for group A patients, and 85.0%, 79.7%, 65.8%, and 65.8% for group B patients, respectively (*p* = 0.282).

A total of nine patients in group A died in the first year, and the causes of death included three AMR, three cases of pneumonia, one heart failure, one hepatocellular carcinoma recurrence, and one hepatic injury during percutaneous transhepatic cholangiographic drainage. A total of four patients in group B died in the first year, and the causes of death included one AMR, one infection with sepsis, one hepatocellular carcinoma recurrence, and one atrial fibrillation with pulmonary emboli. No liver re-transplantation was performed in this study. The 6-month, 1-, 3- and 5-year graft and patient survival were 91.1%, 83.8%, 77.1%, and 71.1% for group A patients, compared with 85.0%, 79.7%, 65.8%, and 65.8% for group B patients, respectively (*p* = 0.282, Figure 5).

### 3.6. Patients with Modified Desensitization Regimen

**Figure 6:**
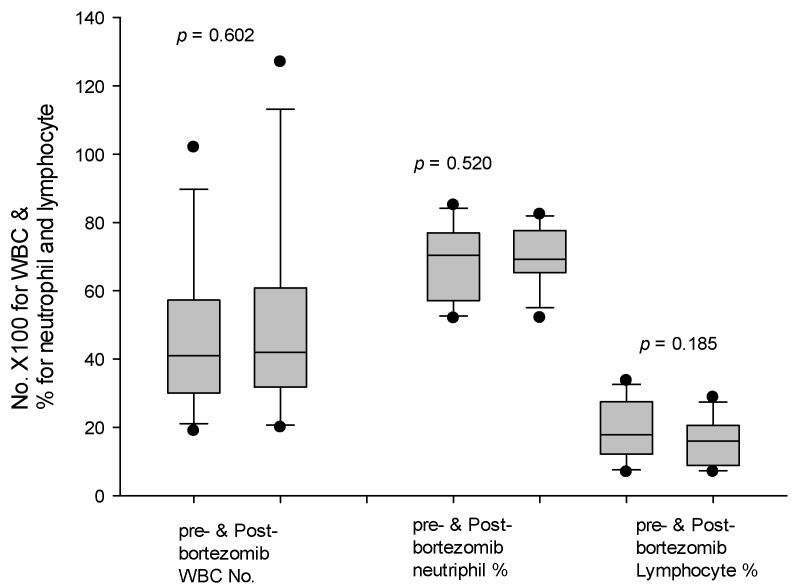
White blood cell count, percentage of neutrophils, and percentage of lymphocytes before and after bortezomib administration. White blood cell count, percentage of neutrophils, and percentage of lymphocytes were not different before and after bortezomib administration.

In the 20 consecutive patients of group B, the last 12 patients received bortezomib-added modified regimen. Among the 12 patients, their median isoagglutinin titers was 1:512 with a range from 1:256 to 1:2048. A total of 7 of the 12 (58.3%) patients experienced postoperative isoagglutinin rebound. However, the isoagglutinin rebound was up to 1:128 only, and no patients developed clinical AMR. Regarding the adverse effects of bortezomib, white blood cell count, percentage of neutrophils, and percentage of lymphocytes before and after bortezomib administration were compared. White blood cell count after bortezomib administration was 5092 ± 2951/mm^3^, compared with 4625 ± 2212/mm^3^ before bortezomib administration (*p* = 0.602). The percentage of neutrophils after bortezomib administration was 70.4 ± 8.7%, compared with 68.0 ± 11.3% before bortezomib administration (*p* = 0.520). The percentage of lymphocytes after bortezomib administration was 15.7 ± 6.9%, compared with 19.5 ± 8.5% before bortezomib administration (*p* = 0.185) (Figure 6). These results revealed that the adverse effects of bortezomib did not appear in this study.

## 4. Discussion

The success of adult ABO-I LDLT is a great progress in liver transplantation, as it overcomes the barrier of blood types [9]. However, adult ABO-I LDLT is still a high risk operation, since the post-operative immune-reactions are more complicated than ABO-compatible liver transplantation. Not only do T-cells mediate acute cellular rejection, but also B-cells contribute to AMR. Therefore, desensitization and immunosuppression procedures were carried to the recipients before transplantation or immediately after transplantation to achieve adult ABO-I liver transplantation [14,21]. Although different preparation regimens for adult ABO-I LDLT are adopted by different centers, rituximab and plasma exchange/plasmapheresis are almost the backbone in all the regimens. Under this desensitization procedure with rituximab and plasma exchange/plasmapheresis, the outcomes of ABO-I LDLT were similar to ABO-compatible liver transplantation [22,23]. However, AMR does occur, and is still an important issue in ABO-I LDLT.

The relevance of isoagglutinin titer to AMR is the most concerning issue in ABO-I liver transplantation. The role of isoagglutinin titers prior to liver transplantation is not determined. In the literature, the patients with initial isoagglutinin titer <1:16 did not develop AMR, although only a simple desensitization protocol was adopted as rituximab only or rituximab with postoperative low dose of intravenous immunoglobulin [24,25]. In this study, the patients with low and high initial isoagglutinin titers were all included, the target titer of isoagglutinin at transplantation was ≤1:64, and the incidence of clinical AMR was 6.6%. The target isoagglutinin titers at transplantation were various among different centers. In a Japanese multicenter study, the results revealed that the incidence of AMR in rituximab era was around 6%, and isoagglutinin titers ≥1:16 at transplantation had a 2.67-fold risk of developing AMR compared to isoagglutinin titers <1:16 in univariate analysis, but did not influence overall survival [21]. In this study, rituximab-based desensitization regimen with isoagglutinin titer ≤1:64 at transplantation could yield the similar results to the Japanese study. Taken together, no AMR occurred in the patients with initial isoagglutinin titer <1:16, even only simple rituximab was given. The outcomes of adult ABO-I LDLT with rituximab desensitization was similar to that of ABO-compatible transplantation [14]; however, the 6% of AMR was not diminished when the patients had initial isoagglutinin titer ≥1:16.

To diminish AMR, post-operative rebound of isoagglutinin should be seriously noted, since postoperative high isoagglutinin titers might cause AMR and result in graft loss. However, determining who will experience isoagglutinin rebound after transplantation is not mentioned often in the literature. In this study, the result showed that the patients with initial high isoagglutinin titers had a higher incidence of isoagglutinin rebound than the patients with low titers. There was no postoperative isoagglutinin rebound if the initial isoagglutinin titer was <1:16, reported by Lee et al. [25]. As only few patients’ isoagglutinin titers are <1:16 at initial assessment, almost all the patients have the possibilities of isoagglutinin rebound. Hence, all the patients with isoagglutinin titers ≥1:16 require desensitization, which is not only for reducing isoagglutinin titers to undergo liver transplantation, but also for preventing postoperative isoagglutinin rebound. Nevertheless, the intensity of isoagglutinin rebound which will trigger clinical AMR is not really known.

Although there is no clear clinical evidence of what titers of postoperative isoagglutinin will cause clinical AMR in ABO-I liver transplantation, it is believed that clinical AMR might be related to the intensity of isoagglutinin rebound. In a Japanese multiple centers’ study, the results showed that peak postoperative IgG isoagglutinin titer ≥1:256 increased 2.5-fold risk of developing clinical AMR, but there was no clear isoagglutinin cutoff level of developing AMR [9]. In fact, the incidence of AMR may be higher than those diagnosed. Haga et al. analyzed C4d immunostaining pattern from 34 ABO-I liver transplant patients with suspected acute rejection, and they found that 17 patients were positive for C4d staining. Their median postoperative isoagglutinin titers was 1:256, and five of them had AMR [16]. AMR would develop sub-clinically, and the risk of clinical AMR was increased when isoagglutinin titer rebound was ≥1:256. In this study, only the five patients with isoagglutinin rebound ≥1:1024 had clinical figures of AMR. By the ROC curve, the specificity of cut-off value of 1:1024 was 1.0. This meant clinical AMR was likely to occur when isoagglutinin rebound was ≥1:1024. Hence, the highest tolerable isoagglutinin titer rebound was 1:512. Clinically, isoagglutinin rebounds suddenly, and the rebound intensity is not predictable. It is better to keep close monitoring on isoagglutinin rebound, and prepare treatment when isoagglutinin titer reaches >1:256.

As we know, antibodies are mainly produced by activated B-cells, memory B-cells and plasma cells, and depletion of both B-cells and plasma cells before transplantation may mitigate isoagglutinin rebound after transplantation. Since B-cells are already depleted by rituximab in current desensitization regimen, bortezomib may be added in the desensitization regimen to deplete plasma cells, particularly in those patients with high isoagglutinin titers. Bortezomib is a proteasome inhibitor, and can induce plasma cell apoptosis [26,27]. We applied bortezomib to deplete plasma cells in our last 12 patients with initial high agglutinin titers. Although 7 of 12 patients experienced isoagglutinin rebound, isoagglutinin titers never rebounded to pre-operative levels to cause clinical AMR. In contrast to these 12 patients, three of the other eight patients in group B had isoagglutinin rebound to high levels, and two (25%) of them developed clinical AMR. Even in the group A patients with low isoagglutinin titers before transplantation, three of them had isoagglutinin rebound beyond 1:512 and developed clinical AMR. Bortezomib is already applied to treat AMR [28,29,30]. In a murine study, bortezomib could eliminate alloantibody-secreting plasma cells and reduce alloantibodies [31]. It was also proposed as a possible agent in a desensitization protocol for highly sensitized patients in kidney transplantation [32,33]. According to the results in this study, bortezomib could prevent isoagglutinin over-rebound after transplantation, and might be considered for addition in the desensitization regimen for ABO-I living donor liver transplantation.

Clinical AMR may be presented as massive hepatocyte necrosis or intrahepatic ischemic cholangitis, which was well described by Tanabe et al. [34]. Both types of AMR are severe complications which lead to graft loss eventually. Re-transplantation is the only way to rescue patients. Re-transplantation is almost impossible in the areas of organ shortage, and prevention of AMR will be the best policy. Depletion of both B-cells and plasma cells may be the ideal way to mitigate postoperative isoagglutinin rebound and avoid clinical AMR. In this study, 12 high-titer patients with additional bortezomib had limited isoagglutinin rebound, and no patients developed clinical AMR. Bortezomib might contribute to mitigate isoagglutinin rebound and avoid clinical AMR.

This study was retrospective to examine the effects of modified desensitized regimen. Although the number of patients receiving modified desensitization regimen were limited, we believe that the data in this study are important to show how to manage the patients with high isoagglutinin titers in ABO-I organ transplantation. AMR is an immune reaction in ABO-I liver transplantation, and some immune complexes may deposit in the liver pathologically. In this study, we only focused on and found the threshold of isoagglutinin rebound to develop clinical AMR. The true incidence of AMR in ABO-I liver transplantation needs further study or to be proven by protocol biopsies.

## 5. Conclusions

Clinical AMR is a high-risk complication in adult ABO-I LDLT. The threshold of isoagglutinin rebound to cause clinical AMR was 1:1024. Rituximab with additional bortezomib into the desensitization regimen could mitigate isoagglutinin rebound and avoid clinical AMR.

## Figures and Tables

**Table 1:** Clinical characteristics of group A and B patients.

	Group A (*n* = 56)	Group B (*n* = 20)	*p*
Gender (M/F)	43/13	16–4	1
Age [median (IQ)]	56.0 (50.5–60.5)	53.4 (48.3–57.8)	0.337
MELD [median (IQ)]	13 (9–18)	16.5 (10–19.5)	0.281
Graft weight (gm)	620 ± 128	662 ± 125	0.209
GRWR [%, median(IQ)]	0.92 (0.80–1.16)	0.99 (0.81–1.16)	0.953
Diseases			
HBV	34	7	
HCV	9	6	
PBC	5		
HBV + HCV	1	6	
Alcoholic	2		
Biliary atresia	1		
Autoimmune	1	1	
idiopathic	3		
HCC (+/−)	30/26	13–7	0.244
Isoagglutinin titer [median (IQ)]	64 (32–128)	512 (256–1024)	<0.001

IQ: interquartile; MELD: model of end stage liver disease; GRWR: graft-to-recipient weight ratio; HBV: Hepatitis B virus; HCV: Hepatitis C virus; PBC: primary biliary cirrhosis; HCC: hepatocellular carcinoma.

## Data Availability

All data analyzed during this study were included in this published article.

## Abbreviations

HCC: hepatocellular carcinoma; BCLC: Barcelona clinic liver cancer; ABO-I: ABO-incompatibility; LDLT: living donor liver transplantation; POD: postoperative day; AST: aspartate aminotransferase; ALT: alanine aminotransferase; CT: computed tomography; ABMR: antibody-mediated rejection.
